# Extraordinary Malformation of the Heart, and Great Arteries of a Child at Birth

**Published:** 1849-03

**Authors:** C. Clark

**Affiliations:** Surgeon, Notting Hill


### RECORD OF MEDICAL SCIENCE.

### ANATOMY AND PHYSIOLOGY.

*Extraordinary Malformation of the heart, and great arteries of a child at birth. By* C. CLARK, *Esq., Surgeon, Notting Hill.*—On the 4th of August, 1847, I was called in to attend Mrs. C——, a fine, healthy young woman, twenty-two years of age, in labour with her first child. The labour was perfectly natural and easy for a first case: she was safely delivered of a female infant, in the course of five or six hours, the only remarkable point about the case being, that the liquor amnii consisted of scarcely more than half a pint of matter somewhat resembling coffee-grounds. The child, when born, seemed puny and feeble, and the respiratory process was not fully established, until the usual means of resuscitation had been adopted, but even then, and afterwards, there remained a lividity about the mouth, and other signs of a weak circulation, though not so remarkable as might be expected, considering the true nature of the cause, of which of course I was ignorant at the time. The mother's breasts being devoid of milk, the child was fed artificially, and on my first visit next morning appeared to be doing pretty well, the excretions having been all duly performed. Next day, however, the nurse stated that it had cried a good deal, and appeared to be griped, for which some cordial medicine was given; but on my visit next day, (the third,) I was informed, that on the previous night it had been seized with a slight convulsive fit, and died almost immediately. Not being able to discover, upon reflection, a sufficient cause of death, even taking into consideration the general feebleness of the constitution, I requested permission to examine the body, which was readily granted, and the result was, the discovery of the following singular malformation. Having submitted the preparation to the inspection of Professor Owen: he has favoured me with the following excellent description, which I beg to quote entire:—

"The exterior form deviates little from that of the normal fœtal heart at birth, but it is rather smaller; the two auricles are indicated by their appendages curving forward towards each other from each side of the base of the heart, and divided by the great arterial trunk answering to the pulmonary artery, but of which the part answering to the ductus arteriosus is continued not only into the descending aorta, but into the ascending portion, giving off the innominata and left subclavian artery. A small vessel like a descending branch from the lower part of the subclavian retrogrades to the interspace of the base of the great arterial trunk, and the right auricle, and appears to furnish, near these, the coronary branches. The appendage of the left auricle is more slender than usual; the shorter and thicker apex of the right auricle has the ordinary size and form. The inner surface of the right auricle shows no prominence between the openings of the superior and inferior venæ cavæ; there is no valve at the orifice of the latter; the Eustachian valve is represented by a very slight ridge. The fossa ovalis covered by a large semi-membranous, semi-muscular valve, with a round orifice at its upper part, leads obliquely into the left auricle. A small fossa at the base of the left auricle, below the entry of the two pulmonary veins, leads into the substance of the thick walls of the single ventricle, but not into its cavity; this is the only indication of a proper auriculo-ventricular aperture; all the blood must have passed directly into the right auricle. The upper part of the common ventricle is crossed obliquely from before, backwards, by a thick muscular arch, arising by several short, fleshy columns, from the fore part of the ventricle, and terminating similarly at its back part. The single arterial trunk arises behind this muscular arch; its orifice is provided with three well developed semilunar valves. The base of the tricuspid valve extends around three-fourths of the right auriculo-ventricular aperture, being wanting on the extreme right side, a large and long membranous valve extends downwards, to be attached to two carneæ columnæ, arising from the apex of the ventricle, forming an incomplete membranous partition, of a semi-cylindrical form, the convexity turning towards the arterial orifice, and separated from it by the muscular arch. The oblique oval outlet of the semi-cylindrical valve opens into the left side of the ventricle, and the blood has then moved along the outside of the cylinder, passing through the large oval aperture, or free space left between its convex side and the anterior wall of the ventricle into the right side of the ventricle from which the great artery takes its origin. Some blood would, however, pass directly from the left half of the cylinder into the right side of the ventricle by a few holes pierced at its lower part, where it becomes attached to the apex of the ventricle, and where it is reduced to a thin lace-work. The pulmonary arteries rise separately from the back part of the common trunk, about three lines above the semilunar valves. The short trunk of the innominata gives off both carotids, as well as the right subclavian, and also a smaller artery, which descends to the interspace of the great arterial trunk and the right auricle, apparently a rudiment of the base of the ascending aorta furnishing the coronary arteries. (Mercury injected into this permeates the coronary branches, and reappears in streams from the cut wall of the ventricle.)

"From the foregoing details it will be seen that the development of the heart has been arrested at a stage corresponding to that of the hearts of serpents, lizards, and turtles—viz., a special auricular cavity for the reception of the blood from the lungs, and another for that from the general system; but both kinds afterwards mixed, and distributed in that state at once to the lungs, and to the general system; but the mode or mechanism by which the pulmonic or systemic venous currents are blended is different from that in those reptiles, the mixture taking place in the right auricle and not in the ventricle. It is more analogous, therefore, to the condition of the auricle in the *Proteus*, according to Rusconi, only that the distinction of the auricles is neither so well marked, externally nor internally, as in this perenni-branchiate batrachian, the partition not being developed. The origin of the pulmonic arteries from a common aortic trunk is also a character peculiar to the batrachian, or lowest reptiles. But the peculiar features of the human fœtal heart, as shown by the single superior cava and the inferior cava; the proportions and shape of the right auricle; the mode of origin of the pulmonic branches and arteries to the head and arms are well marked; and the whole shows an arrested, and, as in the case of the large tricuspid valve, a slightly modified condition of an organ more closely answering to the true human fœtal character of such organ than of that in any of the lower animals which it may resemble in its physiological condition."

I have only, in conclusion, to add, that I have since delivered the mother of a fine healthy boy, which is now living and doing well, and that the father is a stout, healthy little man, so that it is almost impossible to assign a satisfactory reason for such a strange arrest of development.—*London Lancet.*

